# Unveiling the Tapestry of Tobacco Consumption: Exploring the Sociodemographic Factors Impacting Smokers at Smoking Cessation Clinics in Jeddah

**DOI:** 10.7759/cureus.43050

**Published:** 2023-08-06

**Authors:** Ahmad M Alasmari, Sami S Almudarra

**Affiliations:** 1 Preventive Medicine, Ministry of Health (MOH), Riyadh, SAU; 2 Public Health and Epidemiology, Ministry of Health (MOH), Riyadh, SAU

**Keywords:** relapse, quit attempts, medical history, interventions, epidemic, jeddah, smoking-related behaviors, socio-demographic characteristics, smoking cessation, tobacco consumption

## Abstract

Background

The World Health Organization (WHO) has identified tobacco smoking as a global epidemic, causing an estimated three million deaths annually. This study aims to examine the sociodemographic characteristics and smoking-related behaviors among individuals attending smoking cessation clinics in Jeddah during 2022. By identifying these factors, appropriate interventions can be developed to combat the smoking epidemic.

Methodology

The study enrolled male and female participants who visited the Smoking Cessation Clinics in Jeddah from January 2022 to December 2022. Eligible participants were between 18 and 60 years old and agreed to take part in the study. Data on smoking status, medical history, previous attempts at quitting, and medication use were collected. Statistical analysis, including chi-square tests and *P*-values, was conducted to assess the associations between participants' medical history and smoking cessation attempts.

Results

A total of 5,869 participants were included in the study. The findings revealed that approximately one-fifth of the participants had previously attempted to quit smoking, while the majority 4,780 (81.4%) had not made any cessation attempts. Among those who had made quit attempts, the majority had tried quitting between one and four times 968 (16.5%). The duration of successful cessation reported by participants was generally short, with the majority 4,781 (81.5%) not experiencing any extended period of quitting. Common reasons for relapse included cravings, social influences, mood changes, stress, and withdrawal symptoms. The study also found significant associations between specific medical conditions and smoking cessation attempts.

Conclusions

The study identified significant associations between male gender, older age group (51-60 years), divorced marital status, intermediate educational levels, higher income levels, retired status, extreme body mass index (BMI) categories, and previous attempts at smoking cessation. Healthcare providers and policymakers should consider these findings when developing and implementing smoking cessation programs. The insights gained from this research can contribute to the development of targeted interventions to reduce smoking rates and improve public health outcomes.

## Introduction

Tobacco smoking poses a significant global public health challenge, contributing to a range of health issues such as lung cancer, respiratory disorders like chronic obstructive pulmonary disease, eye-related ailments, and arthritis [1]. Additionally, the detrimental effects of smoking extend beyond the individuals who smoke, affecting those near them through exposure to second-hand smoke. Second-hand smoke exposure alone is responsible for causing more than 600,000 deaths each year [2]. According to the World Health Organization (WHO), by 2025, tobacco control initiatives are anticipated to have reduced tobacco prevalence rates throughout all WHO regions [3].

In 2018, the Saudi Food and Drug Authority conducted a survey aimed at updating data on tobacco usage. The results of the survey revealed that 21.4% of the adult population were smokers, which marked a notable increase in prevalence compared to the figure of 12.2% recorded in 2013. This indicates a clear upward trend in smoking rates between the years 2013 and 2018 [4]. Indeed, this continues to be a significant issue as approximately 1.2 million deaths worldwide are attributed to the exposure of nonsmokers to second-hand smoke [1]. Consequently, with the prevalence of current smoking in the Kingdom of Saudi Arabia (KSA) rising from 12.2% in 2013 to 21.4% in 2018, it suggests that there has been a substantial increase in the number of nonsmokers potentially exposed to second-hand smoke.

The Saudi government has made significant efforts to address the issue of tobacco use. In 2005, Saudi Arabia ratified the WHO Framework Convention on Tobacco Control (FCTC) treaty [5].

Additionally, the healthcare system in Saudi Arabia offers complimentary smoking cessation services and a helpline that provides telephonic support and guidance for individuals seeking assistance in quitting smoking. The Ministry of Health (MOH) in Saudi Arabia has established various tobacco cessation centers throughout the country to implement comprehensive smoking cessation programs and treatments. These programs encompass a range of strategies, including medical interventions and behavioral approaches. Medical interventions encompass consultations and the prescription of medications like nicotine replacement therapy (NRT), nicotinic receptor agonists, Varenicline, and antidepressants [6]. Behavioral interventions focus on educating individuals about the significance of smoking cessation and providing psychological support to tobacco smokers through the involvement of healthcare professionals [6].

A better understanding of the sociodemographic characteristics and behavioral factors among visitors to a smoking clinic in Jeddah will help identify specific target groups. Therefore, this study aims to provide up-to-date information on sociodemographic smoking determinants, facilitating the development of appropriate interventional policies to reduce the smoking epidemic.

## Materials and methods

This study was an analytical cross-sectional study conducted at the Ministry of Health smoking cessation clinics in Jeddah, Makkah Province, Saudi Arabia. All participants who visited the Ministry of Health smoking cessation clinics in Jeddah from January 1 to December 31, 2022, were included in the study. The total number of participants during the study period was 5,871. Data regarding smokers registered at the clinics between January 1 and December 31, 2018, were obtained from the National Tobacco Control Program of the Ministry of Health.

The data, provided in a spreadsheet, contained variables related to the smoking clinics, including sociodemographic factors, such as gender, age group, marital status, education, income, and occupation; smoking-related factors, such as the age of smoking initiation, presence of smokers at home, smoking duration, number of packs smoked per day, and quit attempts; and other clinical information, such as the presence of other diseases, medications, etc.

Ethical approval to conduct the study was obtained from the Ministry of Health's institutional review board (IRB) at King Fahd Medical City in Saudi Arabia. Approval was also obtained from the Ministry of Health's Tobacco Control Program in Riyadh. The confidentiality and anonymity of participants' data were preserved, and the data obtained were used solely for this study.

Data analysis was performed using the IBM SPSS Statistics for Windows, Version 23.0 (IBM Corp., Armonk, NY, USA), encompassing both descriptive and inferential statistics. A *P*-value ≤0.05 was considered statistically significant. Frequencies of variables and the relationship between descriptive and outcome variables were examined. Categorical variables were summarized as frequencies and proportions (percentages), while mean values and corresponding standard deviation (SD) values were calculated to summarize continuous variables. Comparisons were made to assess the distributions of selected variables (early smoking initiation, heavy smoking of two packs or more, and smoking duration of above 15 years) among groups defined by sociodemographic characteristics and smoking-related behaviors. Chi-square (*χ*^2^) tests were applied as all the variables were categorical. The *P*-values were reported to indicate the significance of the associations.

## Results

The sociodemographic characteristics of the participants (*n *= 5,869) are presented in Table 1. The majority of participants attended the Jeddah Mobile Clinic 2,795 (47.6%), and a significant proportion of participants were male 5,585 (95.2%). The age distribution indicated that the highest percentages were in the age ranges of 26-30 years 1,169 (19.9%) and 31-40 years 1,606 (27.4%). Regarding marital status, the majority of participants 3,272 (55.8%) were married. Participants exhibited diverse educational backgrounds, with the largest group having a bachelor's degree 2,306 (39.3%). On average, participants started smoking at the age of ±18 years, and the majority 4,515 (76.9%) reported no smokers at home. The majority of participants 4,849 (82.6%) had a smoking period of less than 15 years. Further details can be found in Table 1.

**Table 1:** Sociodemographic characters of the participants (n = 5,869). SAR, Saudi Arabian Riyal

Parameter		*n* (%)
Gender	Female	284 (4.8)
	Male	5585 (95.2)
Age (years)	18-20	249 (4.2)
	20-22	510 (8.7)
	23-25	998 (17)
	26-30	1,169 (19.9)
	31-40	1,606 (27.4)
	41-50	790 (13.5)
	51-60	399 (6.8)
	>60	148 (2.5)
Marital status	Divorce	81 (1.4)
	Married	3,272 (55.8)
	Single	2,503 (42.6)
	Widower	13 (0.2)
Education	Bachelor	2,306 (39.3)
	Diploma	478 (8.1)
	Doctorate	43 (0.7)
	Higher school	1,823 (31.1)
	Illiterate	37 (0.6)
	Intermediate	224 (3.8)
	Master's	85 (1.4)
	Others	748 (12.7)
	Primary	125 (2.1)
Monthly income (SAR)	3,000-5,999	915 (15.6)
	6,000-10,000	1,019 (17.4)
	<3,000	569 (9.7)
	>10,000	658 (11.2)
	No income	2,708 (46.1)
Occupation	Business	198 (3.4)
	Free	810 (13.8)
	Govt. employee	1,449 (24.7)
	Housewife	79 (1.3)
	Others	1,302 (22.2)
	Private sector	857 (14.6)
	Retired	219 (3.7)
	Student	955 (16.3)
Any smoker at home	No	4,515 (76.9)
	Yes	1,354 (23.1)
Smoking period >15 years	No	4,849 (82.6)
	Yes	1,020 (17.4)
BMI (kg/m^2^)		27.11 ± 5.39

Table 2 presents information on smoking cessation attempts and techniques among the participants in Jeddah. The majority of participants 4,780 (81.4%) reported no previous attempts to quit smoking. However, 1,089 (18.6%) of the participants had made previous attempts, with most attempting to quit between one and four times 968 (16.5%). A smaller proportion had made five to nine attempts 82 (1.4%) or 10 or more attempts 39 (0.7%). Regarding the longest period of cessation, the majority of participants 4,781 (81.5%) had not experienced any prolonged period of quitting smoking. Nevertheless, a small percentage reported periods of cessation, with 119 (2%) achieving one to four years of cessation and 368 (6.3%) achieving one to six months of cessation. Only a few participants reported even longer periods of cessation.

**Table 2:** Smoking cessation attempts and techniques among the participants (n = 5,869). NRT, nicotine replacement therapy

Parameter		*n* (%)
Previous attempts of cessation	No	4,780 (81.4)
	Yes	1,089 (18.6)
Number of attempts	None	4,780 (81.4)
	One to four	968 (16.5)
	Five to nine	82 (1.4)
	10 or more	39 (0.7)
Longest period of cessation	None	4,781 (81.5)
	One to four years	119 (2)
	One to six months	368 (6.3)
	Five years or more	29 (0.5)
	Six months to one year	90 (1.5)
	Less than one month	482 (8.2)
Reasons of relapse (if applicable)	Not specified	4,837 (82.7)
	Boredom	14 (0.2)
	Craving	105 (1.8)
	Family problems	8 (0.1)
	Friends	321 (5.5)
	Habit	13 (0.2)
	Medication	5 (0.1)
	Mood changes	164 (2.8)
	Social problems	14 (0.2)
	Stress	331 (5.6)
	Travel	6 (0.1)
	Withdrawal symptoms	19 (0.3)
	Work	13 (0.2)
Without support	No	5,411 (92.2)
	Yes	458 (7.8)
Counseling only	No	5,824 (99.2)
	Yes	45 (0.8)
NRT patch	No	5,844 (99.6)
	Yes	25 (0.4)
NRT lozenge	No	5,865 (99.9)
	Yes	4 (0.1)
Varenicline tablets	No	5,806 (98.9)
	Yes	63 (1.1)

The reasons for relapse varied, with the most commonly reported reasons, including craving 105 (1.8%), influence of friends 321 (5.5%), mood changes 164 (2.8%), stress 331 (5.6%), and withdrawal symptoms 19 (0.3%). Regarding the specific techniques used for smoking cessation, the majority of the participants did not receive counseling from physicians 5,824 (99.2%) and were not supported by healthcare workers 5,411 (92.2%). This higher percentage indicates that most participants have the ability to stop smoking without needing a physician or healthcare provider, reflecting the strong mentality of the participants in the study. In addition, they did not use NRT patches 5,844 (99.6%), lozenges 5,865 (99.9%), or varenicline tablets 5,806 (98.9%).

In Table 3, we examine the association between smoking cessation attempts and sociodemographic characteristics of the participants. The results revealed significant associations with gender (*P *= 0.047), age group (*P *< 0.001), marital status (*P *< 0.001), monthly income (*P *< 0.001), and occupation (*P *< 0.001). Further details can be found in Table 3.

**Table 3:** Attempts of smoking cessation in association with sociodemographic characters of the participants (n = 5,869).

Parameter		Previous attempt of cessation, *n* (%)		*P*-value
		No	Yes	
Gender	Female	244 (85.9)	40 (14.1)	0.047
	Male	4,536 (81.2)	1,049 (18.8)	
Age (years)	18-20	246 (98.8)	3 (1.2)	0.000
	20-22	406 (79.6)	104 (20.4)	
	23-25	861 (86.3)	137 (13.7)	
	26-30	985 (84.3)	184 (15.7)	
	31-40	1,259 (78.4)	347 (21.6)	
	41-50	606 (76.7)	184 (23.3)	
	51-60	300 (75.2)	99 (24.8)	
	>60	117 (79.1)	31 (20.9)	
Marital status	Divorce	60 (74.1)	21 (25.9)	0.000
	Married	2,565 (78.4)	707 (21.6)	
	Single	2,145 (85.7)	358 (14.3)	
	Widower	10 (76.9)	3 (23.1)	
Education	Bachelor	1,891 (82)	415 (18)	0.000
	Diploma	383 (80.1)	95 (19.9)	
	Doctorate	42 (97.7)	1 (2.3)	
	Higher school	1441 (79)	382 (21)	
	Illiterate	33 (89.2)	4 (10.8)	
	Intermediate	162 (72.3)	62 (27.7)	
	Master	68 (80)	17 (20)	
	Others	650 (86.9)	98 (13.1)	
	Primary	110 (88)	15 (12)	
Monthly income (SAR)	3,000-5,999	730 (79.8)	185 (20.2)	0.000
	6,000-10,000	729 (71.5)	290 (28.5)	
	<3,000	460 (80.8)	109 (19.2)	
	>10,000	456 (69.3)	202 (30.7)	
	No income	2,405 (88.8)	303 (11.2)	
Occupation	Business	188 (94.9)	10 (5.1)	0.000
	Free	765 (94.4)	45 (5.6)	
	Govt. employee	1,073 (74.1)	376 (25.9)	
	Housewife	66 (83.5)	13 (16.5)	
	Others	1,081 (83)	221 (17)	
	Private sector	666 (77.7)	191 (22.3)	
	Retired	152 (69.4)	67 (30.6)	
	Student	789 (82.6)	166 (17.4)	
Any smoker at home	No	3,845 (85.2)	670 (14.8)	0.000
	Yes	935 (69.1)	419 (30.9)	
Smoking period >15 years	No	4,196 (86.5)	653 (13.5)	0.000
	Yes	584 (57.3)	436 (42.7)	

Table 4 presents the results of the association between the longest cessation period and sociodemographic characteristics of the participants.

**Table 4:** Longest cessation period in association with sociodemographic characters of the participants (n = 5,869)

Parameter		Longest cessation period, *n* (%)						*X*^2^	*P*-value
		No attempts	Less than one month	One to six months	Six months to one year	One to four years	Five years or more		
Gender	Female	244 (85.9)	20 (7)	14 (4.9)	0 (0)	5 (1.8)	1 (0.4)	6.9	0.229
	Male	4537 (81.2)	462 (8.3)	354 (6.3)	90 (1.6)	114 (2)	28 (0.5)		
Age (years)	18-20	246 (98.8)	1 (0.4)	2 (0.8)	0 (0)	0 (0)	0 (0)	190.4	0.000
	20-22	407 (79.8)	58 (11.4)	35 (6.9)	4 (0.8)	6 (1.2)	0 (0)		
	23-25	861 (86.3)	62 (6.2)	63 (6.3)	3 (0.3)	8 (0.8)	1 (0.1)		
	26-30	985 (84.3)	80 (6.8)	71 (6.1)	20 (1.7)	11 (0.9)	2 (0.2)		
	31-40	1,259 (78.4)	163 (10.1)	100 (6.2)	36 (2.2)	38 (2.4)	10 (0.6)		
	41-50	606 (76.7)	78 (9.9)	47 (5.9)	14 (1.8)	35 (4.4)	10 (1.3)		
	51-60	300 (75.2)	30 (7.5)	39 (9.8)	9 (2.3)	18 (4.5)	3 (0.8)		
	>60	117 (79.1)	10 (6.8)	11 (7.4)	4 (2.7)	3 (2)	3 (2)		
Marital status	Divorce	60 (74.1)	10 (12.3)	5 (6.2)	2 (2.5)	3 (3.7)	1 (1.2)	101.4	0.000
	Married	2,565 (78.4)	293 (9)	225 (6.9)	72 (2.2)	91 (2.8)	26 (0.8)		
	Single	2,146 (85.7)	177 (7.1)	138 (5.5)	16 (0.6)	25 (1)	1 (0)		
	Widower	10 (76.9)	2 (15.4)	0 (0)	0 (0)	0 (0)	1 (7.7)		
Education	Bachelor	1,891 (82)	181 (7.8)	136 (5.9)	39 (1.7)	51 (2.2)	8 (0.3)	79.6	0.000
	Diploma	383 (80.1)	34 (7.1)	35 (7.3)	9 (1.9)	13 (2.7)	4 (0.8)		
	Doctorate	42 (97.7)	1 (2.3)	0 (0)	0 (0)	0 (0)	0 (0)		
	Higher school	1,442 (79.1)	173 (9.5)	135 (7.4)	29 (1.6)	35 (1.9)	9 (0.5)		
	Illiterate	33 (89.2)	2 (5.4)	0 (0)	1 (2.7)	1 (2.7)	0 (0)		
	Intermediate	162 (72.3)	23 (10.3)	24 (10.7)	3 (1.3)	8 (3.6)	4 (1.8)		
	Master	68 (80)	5 (5.9)	7 (8.2)	2 (2.4)	2 (2.4)	1 (1.2)		
	Others	650 (86.9)	59 (7.9)	23 (3.1)	7 (0.9)	6 (0.8)	3 (0.4)		
	Primary	110 (88)	4 (3.2)	8 (6.4)	0 (0)	3 (2.4)	0 (0)		
Monthly Income (SAR)	3,000-5,999	730 (79.8)	87 (9.5)	62 (6.8)	9 (1)	26 (2.8)	1 (0.1)	274.8	0.000
	6,000-10,000	729 (71.5)	119 (11.7)	97 (9.5)	30 (2.9)	32 (3.1)	12 (1.2)		
	<3,000	461 (81)	59 (10.4)	34 (6)	4 (0.7)	11 (1.9)	0 (0)		
	>10,000	456 (69.3)	78 (11.9)	62 (9.4)	24 (3.6)	28 (4.3)	10 (1.5)		
	No income	2405 (88.8)	139 (5.1)	113 (4.2)	23 (0.8)	22 (0.8)	6 (0.2)		
Occupation	Business	188 (94.9)	6 (3)	3 (1.5)	0 (0)	1 (0.5)	0 (0)	244.1	0.000
	Free	765 (94.4)	16 (2)	22 (2.7)	2 (0.2)	5 (0.6)	0 (0)		
	Govt. employee	1073 (74.1)	160 (11)	112 (7.7)	39 (2.7)	50 (3.5)	15 (1)		
	Housewife	66 (83.5)	5 (6.3)	5 (6.3)	1 (1.3)	1 (1.3)	1 (1.3)		
	Others	1081 (83)	105 (8.1)	67 (5.1)	22 (1.7)	21 (1.6)	6 (0.5)		
	Private sector	666 (77.7)	81 (9.5)	70 (8.2)	13 (1.5)	24 (2.8)	3 (0.4)		
	Retired	152 (69.4)	22 (10)	29 (13.2)	4 (1.8)	8 (3.7)	4 (1.8)		
	Student	790 (82.7)	87 (9.1)	60 (6.3)	9 (0.9)	9 (0.9)	0 (0)		
Any smoker at home	No	3845 (85.2)	311 (6.9)	221 (4.9)	54 (1.2)	69 (1.5)	15 (0.3)	182.7	0.000
	Yes	936 (69.1)	171 (12.6)	147 (10.9)	36 (2.7)	50 (3.7)	14 (1)		
Smoking period >15 years	No	4197 (86.6)	307 (6.3)	215 (4.4)	53 (1.1)	62 (1.3)	15 (0.3)	490.9	0.000
	Yes	584 (57.3)	175 (17.2)	153 (15)	37 (3.6)	57 (5.6)	14 (1.4)		

Regarding gender, both female and male participants had a majority of no attempts at smoking cessation. The percentages of participants reporting different cessation periods were similar for both genders. The chi-square test indicated no statistically significant association between gender and the longest cessation period (*P* = 0.229).

For age, participants aged 18 to 20 years had the highest proportion of no attempts at smoking cessation 246 (98.8%). As age increased, the percentages of participants with longer cessation periods also increased. The association between age and the longest cessation period was statistically significant (*P* < 0.001).

Marital status showed a significant association with the longest cessation period. Divorced participants had the lowest percentage of no attempts at cessation 60 (74.1%), while single individuals had the highest percentage of no attempts at cessation 2,146 (85.7%). Widowers had the highest percentage 1 (7.7%) of cessation periods of five years or more. The chi-square test demonstrated a significant relationship between marital status and the longest cessation period (*P* < 0.001).

Education level was significantly associated with the longest cessation period (*P* < 0.001). Participants with a doctorate degree had the highest percentage of no attempts at cessation 42 (97.7%), while those with intermediate education had the highest percentage of cessation periods of five years or more 4 (1.8%). Monthly income also showed a significant association with the longest cessation period (*P* < 0.001). Participants with no income had the highest proportion of no attempts at cessation 2,405 (88.8%), whereas those with an income of more than 10,000 had the highest percentage of cessation periods of five years or more 10 (1.5%).

## Discussion

The study shows that nearly one-fifth of the participants had made previous attempts at cessation of smoking, whereas the majority (4,780, 81.4%) did not attempt to quit smoking. Among those who had made attempts to quit, the majority (968, 16.5%) had made between one to four attempts, indicating that multiple quit attempts may be necessary for successful cessation. These results diverge significantly from the typical recommendations provided by smoking cessation programs, which usually advocate for eight to 14 quit attempts as proposed by organizations such as the American Cancer Society, the Australian Cancer Council, and the Centers for Disease Control [7,8]. Nevertheless, some existent literature supports our findings, suggesting that a notable percentage, ranging between 40% and 52%, may achieve successful cessation on their initial serious attempt [8,9].

Furthermore, the preponderance of males in the sample (5,585, 95.2%) aligns with previous research that has steadily shown higher smoking prevalence among males compared to females as reported by WHO [10] and in Colombia by the National Administrative Department of Statistics [11].

The highest percentages of smokers were in the age groups of 26-40 years. This finding suggests that individuals in their late 20s and early 30s may be at a grave stage for implementing smoking cessation interventions, as they have likely accumulated several years of smoking but are still relatively young and may be motivated to improve their well-being [12]. Marital status appeared to be linked with smoking cessation attempts, with divorced individuals having the lowest percentage of no cessation attempts and widowers displaying longer cessation periods. These findings suggest that individuals experiencing significant life changes, such as divorce or bereavement, may be more motivated to quit smoking or may find it easier to maintain smoking abstinence. On the other hand, single individuals showed the peak percentage of no cessation attempts, indicating a potential area for targeted smoking cessation interventions [3,13].

Education level and monthly income were also significant factors associated with smoking cessation attempts. Participants with higher education and income levels tended to have higher rates of no cessation attempts, while those with intermediate education and moderate-income levels had a higher percentage of long cessation periods. These findings were supported by numerous studies [14,15]. Interestingly, the study revealed that the majority of participants did not receive counseling from physicians or support from healthcare workers, nor did they use NRT patches, lozenges, or varenicline tablets. This suggests that the participants in the study exhibited a strong sense of self-efficacy and were able to attempt quitting without external assistance [16-18].

It is important to acknowledge several limitations of this study. First, the data relied on self-report measures, which are subject to recall bias and social desirability bias. Longitudinal studies are needed to provide a more comprehensive understanding of these associations over time. Furthermore, the study focused on a specific sample population, and the generalizability of the findings to other populations or regions may be limited. Future research should aim to replicate these findings in diverse populations to enhance the external validity of the results.

## Conclusions

The study found significant associations between male gender, older age group (51-60 years), divorced marital status, intermediate educational levels, higher income levels, retired occupation, extreme BMI category, and previous cessation attempts. Healthcare providers and policymakers should consider these findings when designing smoking cessation programs and offering targeted interventions and support for smoking cessation.
